# Recent advances in understanding and managing male infertility

**DOI:** 10.12688/f1000research.17076.1

**Published:** 2019-05-16

**Authors:** Jonathan Fainberg, James A. Kashanian

**Affiliations:** 1 Department of Urology, Weill Cornell Medicine, 525 E. 68th Street, New York, NY, 10011, USA

**Keywords:** Infertility, Male infertility, azoospermia, varicocelectomy, Sperm DNA fragmentation, semen analysis, capacitation, assisted reproductive technology

## Abstract

Infertility is a prevalent condition affecting an estimated 70 million people globally. The World Health Organization estimates that 9% of couples worldwide struggle with fertility issues and that male factor contributes to 50% of the issues. Male infertility has a variety of causes, ranging from genetic mutations to lifestyle choices to medical illnesses or medications. Recent studies examining DNA fragmentation, capacitation, and advanced paternal age have shed light on previously unknown topics. The role of conventional male reproductive surgeries aimed at improving or addressing male factor infertility, such as varicocelectomy and testicular sperm extraction, have recently been studied in an attempt to expand their narrow indications. Despite advances in the understanding of male infertility, idiopathic sperm abnormalities still account for about 30% of male infertility. With current and future efforts examining the molecular and genetic factors responsible for spermatogenesis and fertilization, we may be better able to understand etiologies of male factor infertility and thus improve outcomes for our patients.

## Introduction

Infertility is a prevalent condition affecting an estimated 70 million people globally. The World Health Organization (WHO) estimates that 9% of couples worldwide struggle with fertility issues and that male factor contributes to 50% of the issues. Many genetic and lifestyle factors have been implicated in male infertility; however, about 30% of cases are still thought to be idiopathic. Recent advances in sperm DNA fragmentation, capacitation, and nanotechnology have shed light on these enigmatic causes. Here, we will discuss the epidemiology, causes, and recent advances in the understanding and management of male factor infertility.

## Discussion/analysis of the recent literature

Infertility is a condition that is well recognized by the WHO. Although worldwide prevalence data are lacking, couple infertility is estimated to affect 72.4 million people globally, according to large population surveys. Additionally, the WHO estimates that 9% of couples worldwide struggle with fertility issues and that the male factor accounts for 50% of couple sub-fertility ^1^. More recent US data gathered during interviews from 22,682 men and women who are 15 to 44 years old suggest that as many as 12% of men are sub-fertile ^2^.

Male infertility has a variety of causes, ranging from genetic mutations to lifestyle choices to medical illnesses or medications. Despite advances in the understanding of male infertility, idiopathic sperm abnormalities still account for about 30% of male infertility ^3^. Nonetheless, a variety of medical comorbid conditions have been found to affect semen parameters. To name a few, these include renal disease, liver failure, hemochromatosis, chronic obstructive pulmonary disease, cystic fibrosis, and multiple sclerosis. An Italian study of 2,100 consecutive infertile men examined the relationship between Charlson Comorbidity Index (CCI), semen parameters, and hormonal levels. The study demonstrated that, with an increasing CCI (a marker of poor health), semen parameters deteriorate and follicle-stimulating hormone (FSH) levels rise, suggestive of pituitary compensation in the setting of spermatogenic dysfunction ^4^. The mechanism by which medical conditions may impact fertility includes effects on hormonal levels, impairment of sexual function (including ejaculatory function), or impairment of testicular function/spermatogenesis. By medically optimizing a man’s health, improvements in medical disease status can improve semen parameters, sexual function, and fertility potential ^5^.

For instance, obesity is associated with male infertility, likely because of hormonal changes secondary to excess adipose tissue. In a retrospective multi-institutional cohort study, Bieniek *et al*. demonstrated an inverse relationship between body mass index (BMI) and testosterone, testosterone-to-estradiol ratio, ejaculate volume, sperm concentration, and morphology ^6^. The authors also reported higher rates of azoospermia and oligospermia among obese men (12.7% and 31.7%, respectively) compared with men of normal weight (9.8% and 24.5%) ^6^. Additionally, couples made up of an overweight or obese man with a female partner of normal BMI have increased time to conceive compared with couples with male partners of normal weight ^7^. Couples undergoing assisted reproductive technology (ART), in which the male partner is obese, also have decreased pregnancy rates and increased pregnancy loss, possibly due to higher DNA fragmentation rates in obese men ^8, 9^.

Similarly, studies suggest that male infertility may be an early sign of poor overall health. Not only may infertility be the presenting sign of an underlying medical condition, but men with abnormal semen parameters may be at a higher risk of malignancy. Testicular cancer risk increases up to 20-fold in men with abnormal semen parameters ^10^. This risk even translates to first-degree relatives of men with abnormal semen analyses ^11, 12^. It has also been suggested that male infertility may be related to an increased risk of prostate cancer ^13^. Additionally, when examining claims data for over 76,000 infertile men in the US, investigators found a 49% increased risk of a broad range of cancers compared with controls ^14^. One study found that azoospermic men have a threefold increased risk of all cancers, suggesting that there is a possible shared etiology between azoospermia and cancer development ^15^. Other recent studies have touted the semen analysis as a barometer for overall men’s health, correlating decreasing semen parameters with increased male morbidity and mortality ^16^.

Levine *et al*., in 2017, published a study demonstrating declining sperm concentrations in the US and worldwide ^17^. The etiologies behind these findings of decreasing sperm counts are difficult to pinpoint but may be due in part to increasing rates of overweight and obese men of childbearing age. Additionally, pesticide exposure and illicit drug and tobacco use could be implicated as well, although no causal relationship between these behaviors and decreased sperm parameters currently exists. In recent years, at-home sperm analysis kits have become widely commercially available, allowing men to do a cursory test of their fertility. Most products provide binary (yes/no) results for sperm concentration on the basis of WHO-recommended cutoff values of either 15 or 20 M/mL ^18^. These provide low-cost and accessible options for diagnosis for men who previously may have forgone medical care.

Advanced maternal age has long been known to negatively influence fertility. In many countries, the average age of paternity is rising and increasing reports demonstrate that advanced age similarly affects male fertility. Contemporary evidence confirms that older men have worse semen parameters, poorer reproductive outcomes with unassisted pregnancy, and an increased risk of health problems in their offspring. Specifically, data show that the offspring of older men have an increased risk of neuropsychiatric conditions. Data on the offspring of older fathers, including IQ scores, social skills, and a variety of other health outcomes, are conflicting and need to be studied in greater depth ^19^.

Another recognized cause of male infertility relates to unintended impacts due to medications. Young men may require medications that can decrease fertility and alter the hypogonadal–pituitary–gonadal axis ^20^. These medications include chemotherapeutic agents, psychotropic medications, long-term cortico-steroid use, calcium-channel blockers, alpha-blockers, 5-alpha reductase inhibitors (for androgenic alopecia/male pattern baldness), or testosterone replacement therapy. Frequently, these medications are prescribed without a conversation regarding possible effects on current or future fertility. These medications can alter semen parameters, decrease spermatogenesis, or lead to increased sexual and ejaculatory dysfunction ^21^. It is of the utmost importance to fully review all past, present, and potential future medications in men actively considering conception and those interested in future fertility.

The role of conventional male reproductive surgeries aimed at improving or addressing male factor infertility has recently been studied in an attempt to expand their narrow indications. These surgeries include varicocelectomy and testicular sperm extraction (TESE). Varicoceles, the abnormally dilated and tortuous veins in the pampiniform plexus, are a common cause of male infertility. Varicoceles are present in an estimated 15 to 20% of the general male population and 35 to 40% of infertile men ^22^. The mechanism of action by which a varicocele affects fertility is thought to be related primarily to blood stasis in the scrotum, creating excess heat, which in turn reduces spermatogenesis ^23^. However, there are additional theories of how a varicocele can negatively affect fertility, including metabolite reflux into the testis and increased reactive oxygen species creating sperm DNA damage and hormonal dysregulation ^24^. Varicocelectomy has been shown to improve semen parameters, as demonstrated in a 2011 meta-analysis, which found improvement in sperm concentration as well as total and progressive motility following varicocele repair ^25^. Typically, semen parameters will improve by 3 to 6 months after repair ^26^. Another meta-analysis, from 2016, confirmed that repairing varicoceles prior to ARTs improves pregnancy and live birth rates in oligospermic and azoospermic men ^27^. One recent study of men with non-obstructive azoospermia (NOA) showed an increased return of sperm to the ejaculate following varicocele repair and higher rates of live births when compared with controls with NOA and no varicocele ^28^. Similarly, a 2016 meta-analysis concluded that varicocelectomy in men with NOA and clinical varicocele improved surgical sperm recovery rates ^29^. In 2012, Mansour Ghanaie *et al*. published a randomized control trial examining varicocele repair in couples with recurrent first-trimester miscarriages ^30^. They showed that varicocelectomy significantly improved semen parameters but interestingly also increased pregnancy rates and decreased miscarriage rates significantly ^30^.

TESE has historically been used only for men who have azoospermia. Recently, men with severe oligospermia (sperm concentration below 5 million sperm per milliliter), cryptozoospermia (viable sperm found only under conventional microscopy of centrifuged semen samples), or sperm with high DNA fragmentation rates have been shown to potentially benefit from TESE ^31^. In 2017, Cui *et al*. demonstrated that testicular sperm were superior to ejaculated sperm in men with cryptozoospermia undergoing intracytoplasmic sperm injection (ICSI); pregnancy rates were 53.6% in the TESE group and 33.3% in the ejaculated sperm group ^32^. However, a 2016 meta-analysis did not support these findings ^33^. Another meta-analysis did show that testicular sperm had lower DNA fragmentation rates than ejaculated sperm and that using ICSI had higher clinical pregnancy and live birth rates ^34^. Further studies are warranted in order to corroborate these results.

Sperm DNA fragmentation is a novel and potentially valuable tool for male fertility evaluation. Increased sperm DNA fragmentation is known to negatively impact pregnancy rates ^35^. Recently, there has been some controversy over the utility of DNA fragmentation tests in predicting ART outcomes. Owing to the lack of standardization among the tests and the inability in smaller studies to predict outcomes, prior guidelines had cautioned practitioners in testing for sperm DNA damage ^36^. However, Simon *et al*. recently published a systematic review and meta-analysis concluding that DNA damage has a negative effect on clinical pregnancy rates following both *in vitro* fertilization and ICSI ^37^. Newer guidelines based on up-to-date evidence regarding these DNA fragmentation tests are now available ^38^.

Given the data on DNA fragmentation, many studies have attempted to identify efficient and effective means of sperm cell sorting to identify the undamaged sperm and selectively use these for ART. Magnetic activated, flow cytometric, and microfluidic sperm sorting are examples of techniques for identifying semen samples with viable sperm low DNA fragmentation indexes ^39– 42^. These techniques have limitations, and research investigating whether nanotechnology can aide in sperm sorting is under way ^43^.

An area that has recently regained attention is capacitation, the functional maturation of sperm that takes place *in vivo* along the female reproductive tract ^44^. As sperm progress toward the egg, sperm respond to stimuli and undergo molecular reactions that prepare them for fertilization. Defects in capacitation impair the fertilizing capability of sperm. The Cap-Score™ is an investigational test that measures sperm capacitation potential ^45^. Other diagnostic tests, such as MiOXSYS, which measures oxidative stress, have also gained interest recently ^46^. Further development of novel semen and sperm tests will aid in providing more dynamic information than the standard semen analysis.

## Conclusions

Infertility is a prevalent condition that affects over 70 million people globally. A variety of lifestyle choices and genetic issues have been implicated in the condition. While poor overall health contributes to infertility, it has also been demonstrated that infertility is associated with an increased risk of a variety of malignancies. Recent studies examining DNA fragmentation, capacitation, and advanced paternal age have shed light on previously unknown topics. Despite recent advances, about one third of cases remain idiopathic. With current and future efforts examining the molecular and genetic factors responsible for spermatogenesis and fertilization, we may be better able to understand etiologies of male factor infertility and thus improve outcomes for our patients.
